# Discrimination of distinct chicken M cell subsets based on CSF1R expression

**DOI:** 10.1038/s41598-024-59368-x

**Published:** 2024-04-16

**Authors:** Safieh Zeinali, Kate Sutton, Masoud Ghaderi Zefreh, Neil Mabbott, Lonneke Vervelde

**Affiliations:** 1 Division of Immunology, The Roslin Institute & Royal (Dick) School of Veterinary Studies, University of Edinburgh, Edinburgh, EH25 9RG UK; 2 Division of Genetics and Genomics, The Roslin Institute & Royal (Dick) School of Veterinary Studies, University of Edinburgh, Edinburgh, EH25 9RG UK

**Keywords:** Chicken, Follicle-associated epithelium, Interfollicular epithelium, M cells, RNA-seq, Cell biology, Immunology

## Abstract

In mammals, a subset of follicle-associated epithelial (FAE) cells, known as M cells, conduct the transcytosis of antigens across the epithelium into the underlying lymphoid tissues. We previously revealed that M cells in the FAE of the chicken lung, bursa of Fabricius (bursa), and caecum based on the expression of *CSF1R*. Here, we applied RNA-seq analysis on highly enriched *CSF1R*-expressing bursal M cells to investigate their transcriptome and identify novel chicken M cell-associated genes. Our data show that, like mammalian M cells, those in the FAE of the chicken bursa also express *SOX8*, *MARCKSL1*, *TNFAIP2* and *PRNP*. Immunohistochemical analysis also confirmed the expression of SOX8 in *CSF1R*-expressing cells in the lung, bursa, and caecum. However, we found that many other mammalian M cell-associated genes such as *SPIB* and *GP2* were not expressed by chicken M cells or represented in the chicken genome. Instead, we show bursal M cells express high levels of related genes such as *SPI1*. Whereas our data show that bursal M cells expressed *CSF1R*-highly, the M cells in the small intestine lacked *CSF1R* and both expressed SOX8. This study offers insights into the transcriptome of chicken M cells, revealing the expression of CSF1R in M cells is tissue-specific.

## Introduction

The mucosa-associated lymphoid tissues (MALT) constitute the primary line of defence against pathogens, infiltrating the body via the mucosal linings of the respiratory, digestive, ocular, and reproductive tracts^1^. Immune tolerance or activation is achieved when antigens are transported across the epithelial cell layer to immune cells in the underlying lamina propria^2^. Avian species have specific MALT organs including the bursa of Fabricius (bursa), Meckel diverticulum, and caecal tonsils, and numerous isolated and aggregated lymphoid follicles are present throughout the intestine^3–6^. Similar to mammals, chicken Peyer’s patches (PP) in the small intestine contain organised lymphoid tissues with B cell-follicles, T-cell areas, and a specialised follicle-associated epithelium (FAE) that contains M cells covering the subepithelial dome region^7^. M cells are a unique population of epithelial cells that specialise in the endocytic uptake and transfer of particulate antigens across the epithelial cell layer to the underlying lymphocytes and mononuclear phagocytes in the subepithelial dome region. M cells have a major role in mucosal responses as demonstrated in mice deficient in M cells which have reduced PP and germinal centre reactions and IgA levels^8,9^.

In mouse, the epithelial cells in the intestine derive from Lgr5-expressing intestinal stem cells in the crypts. Daughter cells derived from these intestinal stem cells differentiate into M cells when stimulated with the cytokine receptor-activator of NF-ĸB ligand (RANKL) expressed by subepithelial dome stromal cells^8,10^. As the immature M cells migrate from the crypt base upward along the crypt–FAE axis, they are detectable by the expression of Annexin A5 (Anxa5) and Myristoylated alanine-rich C-kinase substrate like 1 (Marcksl1)^11,12^. At the late stages of development, the expression of transcription factors belonging to E26 transformation-specific (ETS) and sex-determining region Y (SRY)-related high mobility group (HMG)-box families, SPIB and SRY-box transcription factor 8 (SOX8) are required for their subsequent differentiation into functionally mature Glycoprotein 2 (GP2) expressing M cells^13,14^. The involvement of GP2 and β1-integrin as uptake receptors on M cells has been shown for *Salmonella* and *Yersinia* through their FimH and Invasion proteins, respectively^15,16^.

The bursa represents a lymphoid organ primarily involved in chicken B cell development and is located dorsal to the rectum communicating with the posterior region of the cloaca by a short duct^17^. Morphologically, the bursa consists of numerous lymphoid follicles, each overlayed with FAE cells that contain M cells, surrounded by interfollicular epithelial (IFE) cells ^17^. The study of chicken M cells primarily relied on their ultrastructural characteristics and binding patterns of lectins^18–20^. However, a comparative transcriptome analysis between murine PP-derived and chicken bursa-derived FAE cells revealed *CLU*, *ANXA10*, and Prion protein (*PRNP*) expression were conserved between the two species^21^. In mice, colony stimulating factor 1 receptor (*CSF1R*)-expression is restricted to mononuclear phagocytes and absent in the FAE overlying the PP^22^. In contrast, studies using *CSF1R*-transgene reporter chickens showed that in addition to the mononuclear phagocytes, the FAE cells within the bronchus-associated lymphoid tissue (BALT), bursa, and the caecal tonsils and proctodaeum of the large intestine express *CSF1R*^23–25^. Functionally, these bursal *CSF1R*-transgene^POS^ FAE cells resembled mammalian antigen-sampling M cells^25^. The expression of *CSF1R* by chicken M cells implied that they may differ from their mammalian counterparts in terms of their development and transcriptional regulation. Here, by employing RNA-seq analysis on highly enriched bursal cluster of differentiation (CD) 45^NEG^ *CSF1R*-transgene^POS^ M cells, we show the conservation of expression of several murine M cell-associated genes in bursal M cells while also identifying genes unique to chicken M cells. Although M cells were present in the FAE of the small intestinal PP, these cells lacked expression of CSF1R mRNA and protein. Our data suggest that chickens exhibit diversity in their M cells that differ based on their anatomical location and expression of CSF1R.

## Results

### Phenotypic characterisation of CSF1R-expressing bursal M cells by flow cytometry

**Figure 1:**
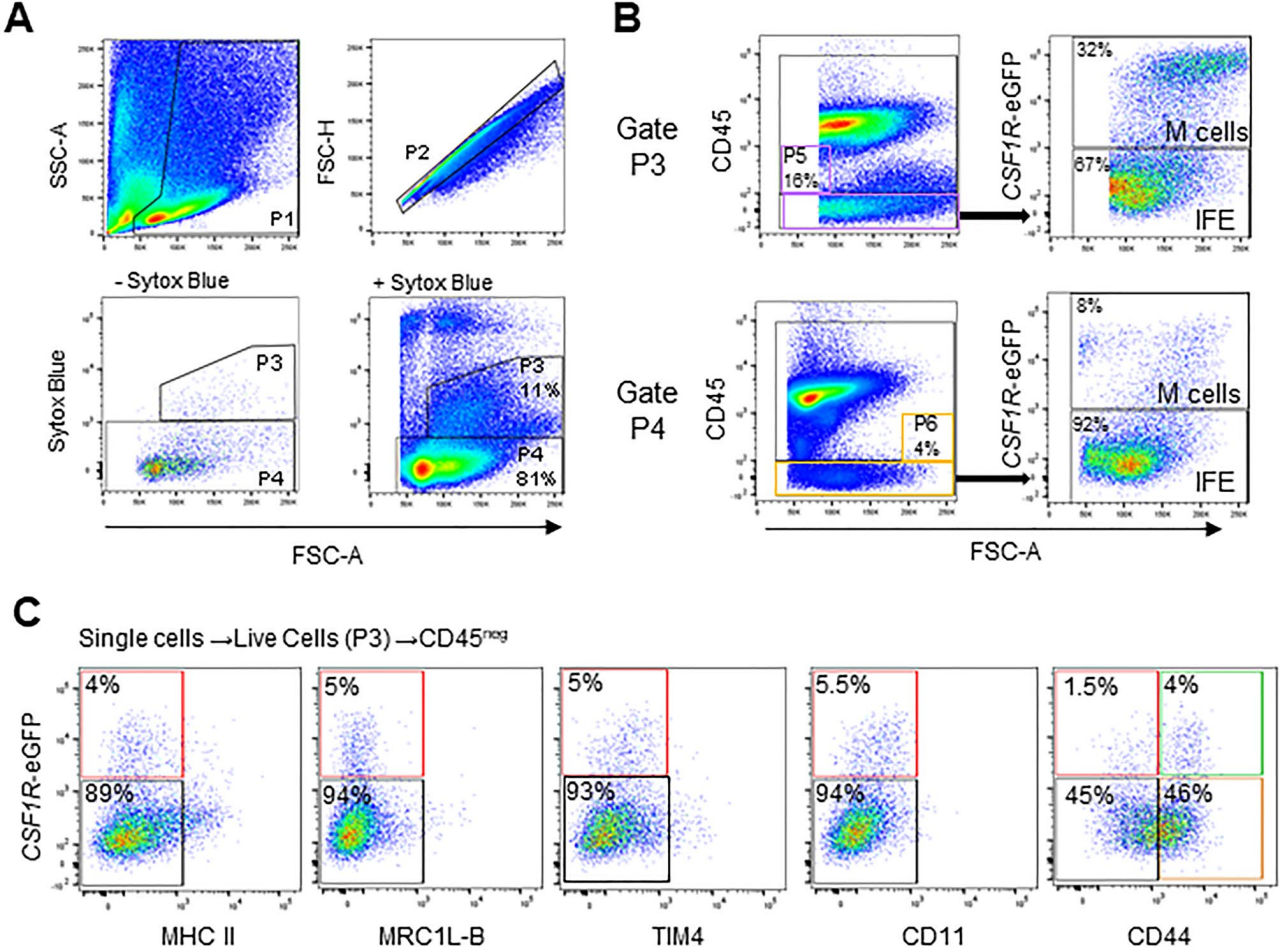
Flow cytometric analysis of isolated chicken bursal cells. (**A**) After debris removal (Gate P1), isolated bursal cells were gated on single cells (Gate P2). Live cells (Sytox Blue^NEG^) were gated based on auto-fluorescence (Gate P3) or negative auto-fluorescence (Gate P4) in the ultraviolet channel. (**B**) To exclude leukocytes, CD45^NEG^ gates were applied to Gates P3 and 4 and analysed for *CSF1R*-eGFP^POS^ cells as putative bursal M cells and *CSF1R*-eGFP^NEG^ cells as putative IFE cells (Gates P5 and P6). Data is representative of 5 biological replicates from 1-week-old *CSF1R*-eGFP reporter transgenic chickens. (**C**) CD45^NEG^ *CSF1R*-eGFP^POS^ M cells lack the surface expression of chicken monocyte/macrophage markers, MHC II, MRC1L-B, CD11, TIM4 while consist of both CD44^POS^ and CD44^NEG^ populations. Data is representative of two independent experiments from 2-week-old *CSF1R*-eGFP reporter transgenic chickens.

*CSF1R*-transgene expression distinguishes the bursal FAE from IFE^24^. In *CSF1R*-reporter transgenic chickens, M cells in bursal FAE express *CSF1R*-transgene and lack the expression of CD45^24^. We utilised the *CSFR-*reporter transgenic chickens to examine the phenotype of bursal *CSF1R*-trangene^POS^ FAE cells (M cells) and *CSF1R*-trangene^NEG^ IFE cells. After gating the bursal cells based on size/granularity and the removal of doublets, and dead cells (Gates P1-2), two subpopulations were observed in the live cell channel (Fig. 1A). Cells within Gate P3 exhibited auto-fluorescence while cells in Gate P4 were low/negative in the violet channel (Fig. 1A). Cells expressing CD45 were excluded from the cells within Gates 3 and 4 to remove leukocytes from the analysis (Fig. 1B). The cells in Gate P3, consisted of 16% of CD45^NEG^ cells compared to 4% in Gate P4. Next, we examined *CSF1R*-eGFP expression within the CD45^NEG^ subpopulations (Gates P5 and P6), and found 32% of the cells in Gate P5 were *CSF1R*-eGFP^POS^ compared to 8% in Gate P6 (Fig. 1B). Based on the CD45^NEG^ *CSF1R*-eGFP^POS^ phenotype these cells represent chicken M cells. A vast majority of cells in the CD45^NEG^ gates (Gates P5 and 6) were also negative for *CSF1R*-eGFP expression and were considered to represent IFE cells. This gating strategy confirmed the ability to isolate populations of live CD45^NEG^ *CSF1R*-eGFP^POS^ M cells and CD45^NEG^ *CSF1R*-eGFP^NEG^ IFE cells from the bursa.

Further phenotypic analysis of CD45^NEG^ *CSF1R*-eGFP^POS^ M cells demonstrated that they lack surface expression of MHC II and chicken monocyte/macrophage markers including MRC1L-B, TIM4, and putative CD11(Fig. 1C). CD44 has been reported as a putative pan-M cell marker^21,24^. CD44 staining of bursal M cells demonstrated heterogenous expression of this marker with two subpopulations, CD44^NEG^ and CD44^POS^. The absence of immune cell markers confirmed that these populations of bursal CD45^NEG^ *CSF1R*-eGFP^POS^ cells and CD45^NEG^ *CSF1R*-eGFP^NEG^ cells were highly enriched in bursal M cells and IFE cells, respectively.

### Stable transcriptional profile of bursal M cells during the post-hatch period

**Figure 2:**
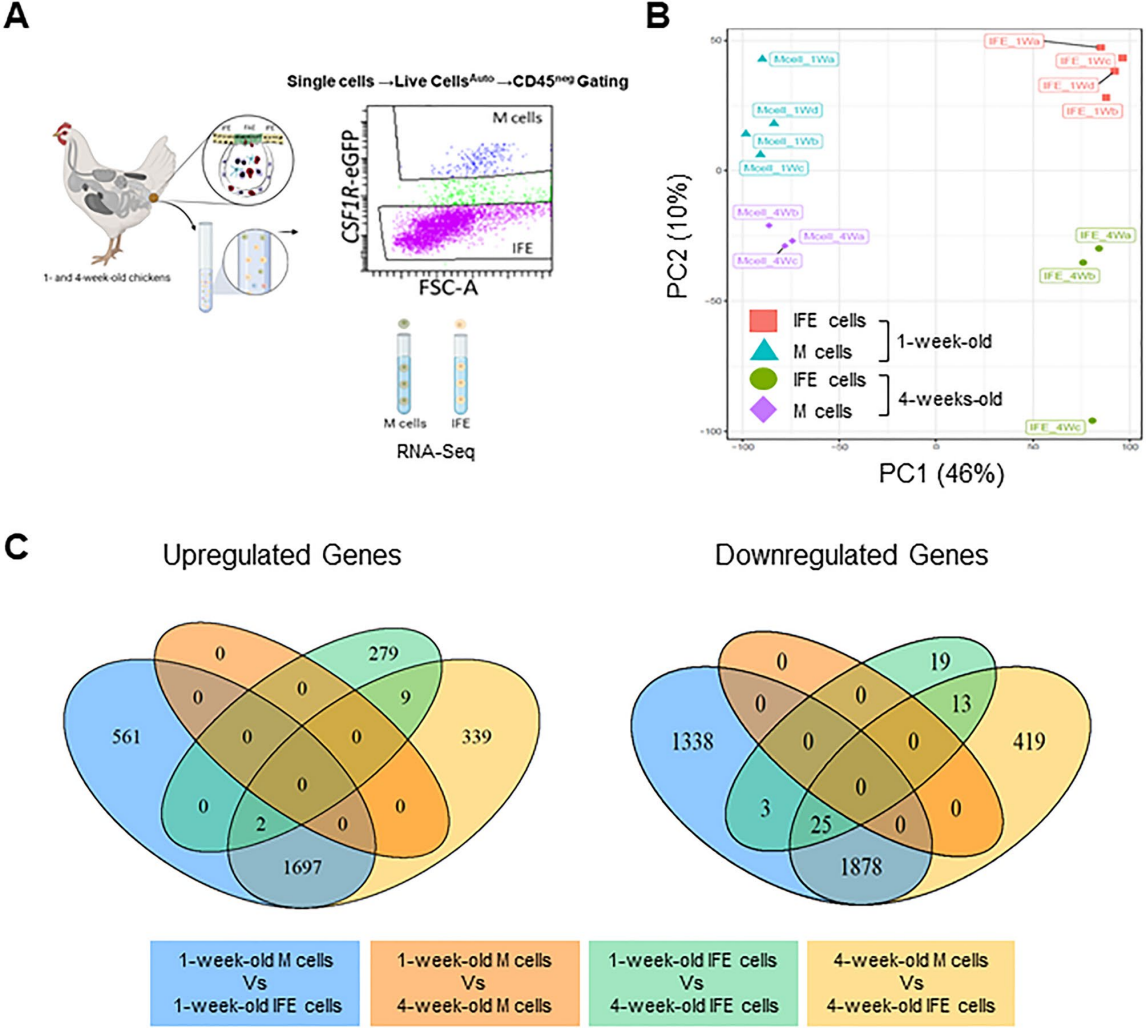
Gene expression profiling of chicken bursal M cells. (**A**) Representative diagram illustrating the isolation and FAC sorting of bursal single, live auto-fluorescent, CD45^NEG^ *CSF1R*-eGFP^POS^ M cells and *CSF1R*-eGFP^NEG^ IFE cells from 1- and 4-week-old chickens for RNA-seq analysis. (**B**) PCA demonstrates the association of sample clustering with the cell type (PC1) and age (PC2). (**C**) Venn diagram demonstrating the number of genes up or downregulated in comparison groups based on cell types (M cells and IFE cells) and age (1- and 4-week-old chickens). DEG were based on FDR < 0.05 with log_2_ FC > 1 for up-regulated and Log_2_ FC < − 1 for down-regulated genes.

At the time of hatch, a bursal follicle segregates into defined cortical and medullary regions while the FAE forms epithelial tufts that transport particulate antigens from the lumen into the bursal follicle^26^. To identify the gene expression profile and assess whether the post-hatch development of the bursa and exposure to gut-derived molecules affect the transcriptome of M cells and IFE cells, we isolated these cells from 1- and 4-week-old chickens. Using a similar flow cytometric gating strategy (Fig. 1A), CD45^NEG^ *CSF1R*-eGFP^POS^ M cells and CD45^NEG^*CSF1R*-eGFP^NEG^ IFE cells were FAC sorted and transcriptome analysis performed by mRNA-seq (Fig. 2A). PC analysis of the global transcriptional profiles of each mRNA-seq dataset identified distinct clusters of M cells and IFE cells where the cell-type difference accounted for 46% and age 10% of the variance in the data (Fig. 2B; PC1, PC2 respectively).

We next compared the number of differentially expressed genes (DEGs) between the age-matched M cells and IFE cells, and the influence of age within each cell population. Analysis of cells from 1-week-old birds revealed 2260 upregulated and 3246 downregulated DEGs in M cells compared to IFE cells (Fig. 2C), whereas analysis of cells from 4-week-old birds revealed 2047 upregulated and 2335 downregulated DEGs in M cells compared to IFE cells (Fig. 2C). Comparison of the transcriptomes between M cells enriched from 1- and 4-week-old chickens found no DEGs that reached the cut-off threshold (FDR < 0.05, log_2_ FC > 1 or < − 1, Fig. 2C). This implied that 1- and 4-week-old M cells share a similar transcriptional profile. In contrast, a similar comparison in the global transcriptomes of IFE cells identified 290 upregulated and 60 downregulated DEGs in 1-week-old IFE cells compared to IFE cells from 4-week-old birds. Together, this analysis suggests that the transcriptional profiles of bursal M cells are very stable during the first four weeks of post-hatch development, whereas changes in the transcriptional profiles of IFE may suggest a potential maturation of the cells.

### Transcriptional profile of bursal M cells differs from murine M cells

To gain insights into the fundamental biology of bursal M cells, we searched upregulated DEGs for key regulatory genes and pathways involved in M cell development and function. With the stability between 1 and 4-week-old M cell transcriptome, we utilised the 1-week-old DEG dataset for further analysis. This data set contains 2260 upregulated and 3246 downregulated DEGs. Functional enrichment analysis of the downregulated DEGs of bursal M cells indicated that the genes upregulated in IFE are involved in pathways related to importing nutrients and their digestion, cell division and DNA replication in line with the IFE being composed of columnar epithelial cells and mucus-producing cells (data not shown). Here we focused on the upregulated DEGs in chicken bursal M cells and compared the genes with a previous study that identified a set of 28 M cell-associated genes conserved across murine PP and chicken FAE cells^21^. The analysis of the 2260 upregulated DEGs identified in 1-week-old M cells compared to IFE cells found that 13 of the 28 genes, originally identified by Nakato et al.^21^, were represented in our dataset (Supplementary Table 1). However, of the missing genes, such as *CLU* and *ANXA10,* have been implicated in mammalian and chicken M cell biology^21,24,27^ while genes not present in our DEG dataset, such as *FLI1*, *CXCR4* and *VIM,* implied that our enrichment approach excluded immune cells and fibroblasts^28,29^.

We next analysed the presence of murine M cell-associated genes within the 2260 upregulated DEGs^12–14,21,30–36^. Although some genes were conserved between murine and chicken M cells, including *Nfkb2*, *Tnfaip2*, *Marcksl1*, TNF receptor superfamily member 11A (*Tnfrsf11a*)*, Tnfrsf11b*, *Relb*, and *Sox8,* other genes such as ETS transcription factor Spi-B (*Spib*), *Gp2*, allograft inflammatory factor 1 (*Aif1*)*, Pglyrp1*, and *Ccl9* are not present in the chicken genome (Table 1 and Supplementary Files 1–2). For example, whereas the expression of *Spib* is essential for the functional differentiation of M cells in the mouse intestine^37^, this gene is currently not annotated in the chicken genome. By searching 2260 upregulated genes of M cells for other subfamily of ETS transcription factors, we found SPI1 was represented (Supplementary File 1–2), implying a similar role in chicken M cells.

**Table 1:**
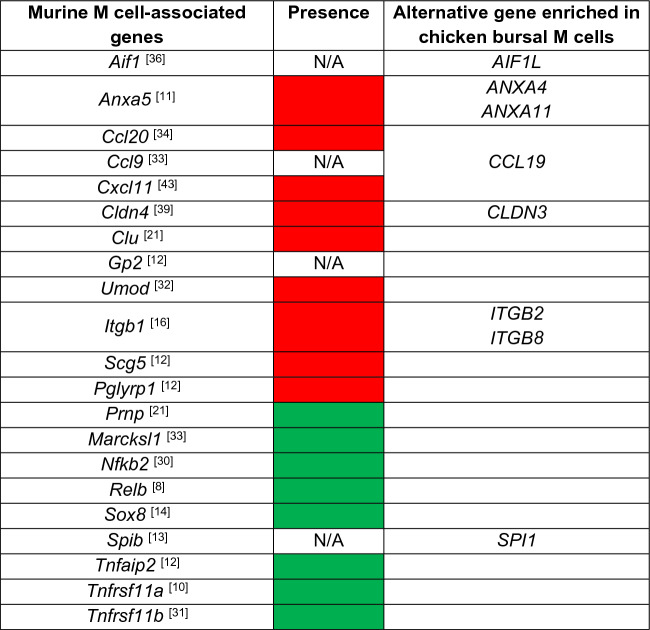
Assessment of the transcriptome of sorted *CSF1R*-transgene^POS^ bursal M cells for the presence (green) and absence (red) of conserved murine M cell-associated genes and alternative related gene family members for non-annotated genes (N/A) in GRCg6a chicken genome and genes not upregulated in chicken M cells.

**Figure 3:**
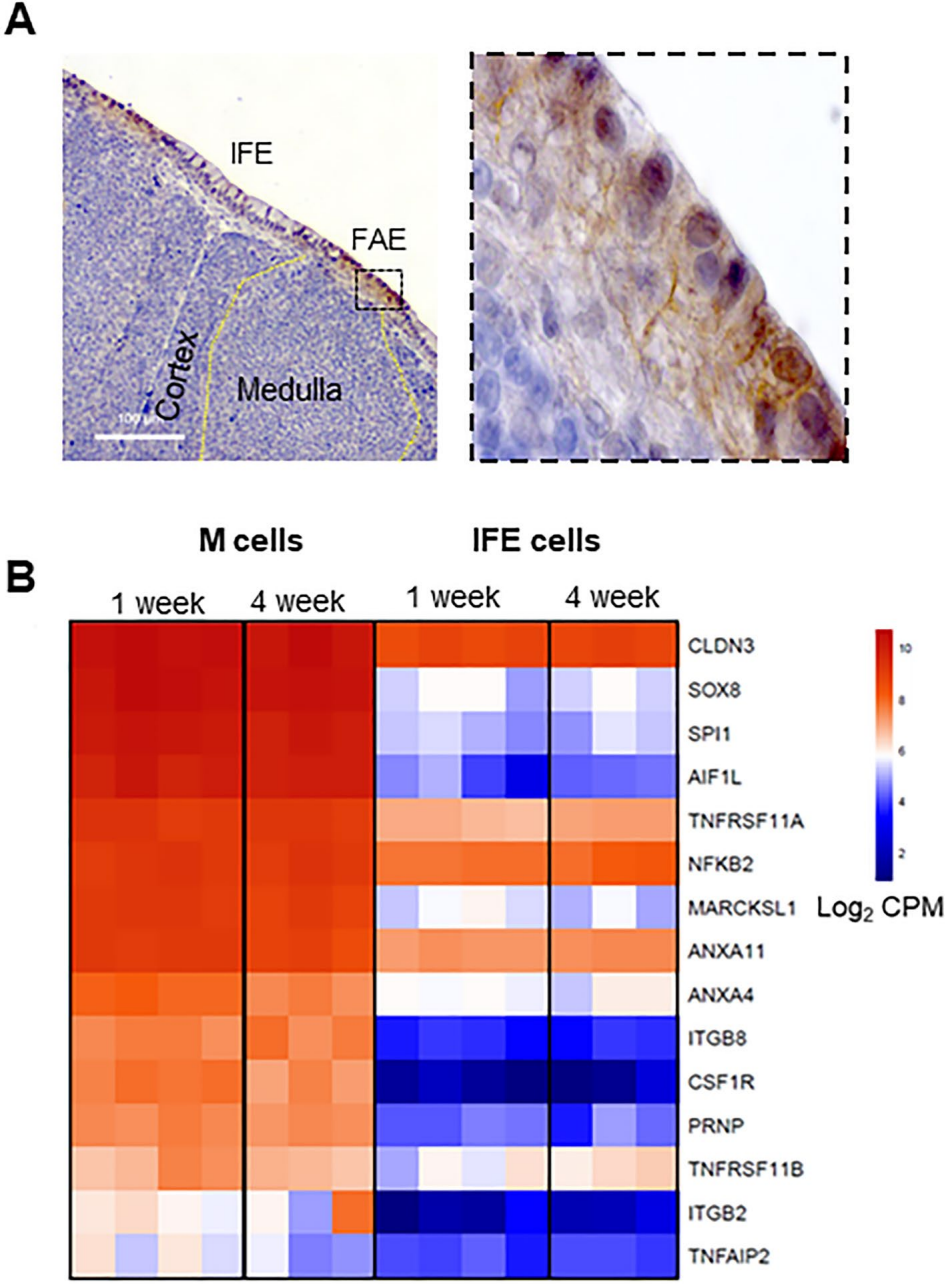
Enrichment of mouse M cell-associated genes in bursal M cells. (**A**) FFPE bursal sections were stained with rabbit anti-human AIF1L (brown) and haematoxylin (blue). Data is representative of four independent experiments using 4-week-old *CSF1R*-eGFP reporter transgenic chickens. Scale bars = 100 and 10 µm. (**B**) Heatmap analysis of the expression levels of murine M cell-associated genes in FAC sorted bursal M cells and IFE cells from 1- and 4-week-old chickens. Genes were identified based on FDR < 0.05 with log_2_ FC > 1 for upregulated and log_2_ FC < − 1 for downregulated genes between M cells and IFE cells of 1-week-old chickens. Data represent n = 4 for 1-week-old and n = 3 for 4-week-old chickens. Heatmaps colouring shows the relative expression level log_2_ CPM (counts per million) of genes (rows) across cells (columns) with red indicating high counts and blue indicating low counts.

A similar approach was applied to other murine M cell-associated genes that were missing from the current chicken genome (GRCg6a) to identify closely-related family members amongst our list of 2260 chicken M-cell upregulated DEGs (Supplementary File 1). This suggested that the genes encoding two annexin family members ANXA5 and ANXA10, were not represented in the chicken M cell DEGs although ANXA10 has been reported to be expressed in chicken and murine M cells^21,24,35^. However, the genes encoding two other annexin family members, *ANXA4* and *ANXA11,* were upregulated. β1 integrin (encoded by *Itgb1*) is an adhesion molecule that is expressed at the basolateral surface of small intestinal enterocytes^38^ and relocated to the apical surface of M cells during *Yersinia enterocolitica* infection and interacts with invasin leading to bacterial uptake through micropinocytosis^16^. *Itgb1* was not present in our dataset but fellow integrin family members integrin β8 (*ITGB8*) and integrin β2 (*ITGB2*) were present (Table 1). A similar observation was made for the tight junction protein CLD4^39^ which was not present, but a close family member *CLDN3* was detected. *Aif1,* is not currently annotated in the chicken genome but a similar gene, allograft inflammatory factor 1 like (*AIF1L*)^40^, was present in the chicken M cell dataset and its expression in M cells of the bursal FAE was confirmed by immunohistochemical staining (Fig. 3A). Both *GP2* and Uromodulin (*UMOD*) are expressed by mature mouse M cells and considered as uptake receptors for certain bacterial species^15,41^. At the time of writing (January 2024), the gene encoding *GP2* was not annotated in the GRCg6a build of the chicken genome and *UMOD* was not present in the dataset. The chemokine genes, chemokine (C–C motif) ligand 9 (*Ccl9*)*, Ccl20* and *Cxcl11*, are highly expressed in the FAE of mice, working to recruit leukocytes^34,42,43^. While the expression of *CCL20* was downregulated in bursal M cells, *CCL9* and *CXCL11* are not annotated in chicken genome. Instead, *CCL19* was the highest upregulated chemokine in our dataset (Supplementary File 1–2) which may function to attract CCR7 expressing macrophages and dendritic cells (DC)^44^. The expression levels of identified M cell-associated genes (Supplementary Table 1) were similar in bursal M cells derived from 1- and 4-week-old chickens (Fig. 3B). To further explore conserved M cell-associated genes in mouse and chicken, we compared the upregulated DEGs identified from single cell RNA-seq data of mouse intestinal M cells^45^ with those of 1-week-old bursal M cells, resulting in the identification of 9 conserved genes (Supplementary Table 2). Taken together, we have identified the conserved expression of some murine M-cell related genes in chicken M cells, including expression of the key transcriptional regulator SOX8^14^. While some mouse M cell-associated genes, such as those encoding GP2 were not represented in the chicken genome, we identified a set of close family members of other mouse M cell associated-genes that were also highly expressed in chicken M cells. However, this analysis also identified many genes such as the transcription factor *SPI1* that were highly expressed in chicken M cells, but not mouse M cells.

### Identification of candidate transcription factor encoding genes involved in bursal M cell development

**Figure 4:**
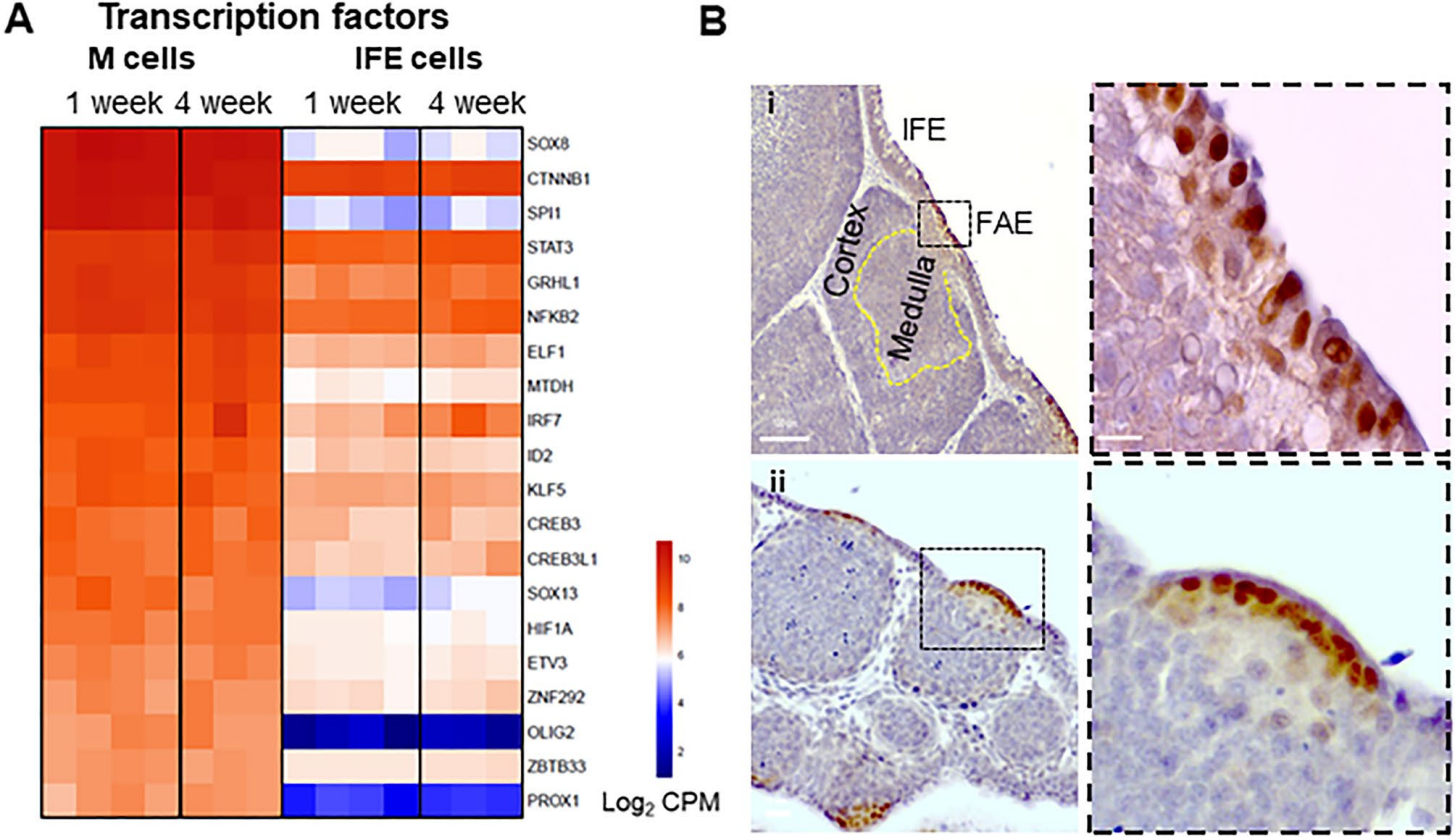
Identification of transcription factors, signalling receptors and putative functional molecules associated with bursal M cells. (**A**) The top 20 upregulated transcription factors and signalling receptors (associated with ‘DNA-binding transcription factor activity’ (GO:0003700) and ‘cytokine-mediated signalling pathway’ (GO:0019221) GO terms, respectively) in sorted bursal M cells and IFE cells from 1- and 4-week-old chickens. (**B**) FFPE bursal sections stained with rabbit anti-human SOX8 (brown) and haematoxylin (blue). Data is representative of at least four independent experiments using (i) 4-week-old *CSF1R-*reporter transgenic chickens and (ii) 18-day-old embryos. Scale bars = 100 and 10 µm. Heatmaps colouring shows the relative expression level log_2_ CPM (counts per million) of genes (rows) across cells (columns) with red indicating high counts and blue indicating low counts.

We next searched for the presence of other transcriptional regulators that may provide insights into the differentiation and functional maturation of these cells. Filtering this list using the ‘DNA-binding transcription factor activity’ GO term (GO:0003700) identified transcription factors, belonging to the following families; winged helix/forkhead (*ELF1, ELF5, ETV3, IRF7, SPI1*); HMG box (*SOX8*, *SOX13*, *SOX21*); DNA-binding (*BBX, ID2*, *STAT3*); C2H2 zinc finger (*KLF5, KLF9, ZNF292*, *ZBTB33*); and basic helix-loop-helix (*ASCL2*, *HIF1A, OLIG2*) (Supplementary File 3). Analysis of top 20 highly expressed transcription factors in M cells compared to IFE cells revealed two transcription factors, *SOX8* (conserved in murine M cells^14^), and *SPI1* (associated with chicken M cells), highly enriched in bursal M cells (Fig. 4A). Immunostaining using an anti-human SOX8 antibody confirmed the specific expression of SOX8 protein in chicken bursal M cells (Fig. 4B). Although *CTNNB1* was the second highest gene in chicken M cells, it was also highly expressed in IFE cells and thus unlikely to have a specific role in chicken M-cell development. Since *SPI1* expression was unique to chicken M cells, we used gProfiler to determine whether the chicken M cell genes were enriched in binding site motif for this transcription factor (Supplementary File 4). This analysis suggested that 472 (~ 20%) genes have promoter regions with SPI1 binding motifs, indicating an important role of *SPI1* in M cell biology. RANKL-RANK signal through the NFκB pathway which is essential for M cell differentiation in mice^30,46,47^. NFκB binding site motifs were present in the promoter regions of 1293 (57%) DEGs in chicken M cells (Supplementary File 4), indicating the conserved requirement of this pathway for M cell differentiation in mice and chickens. This analysis supports the hypothesis that the chicken CSF1R^POS^ M cells have a distinct transcriptional profile compared to murine M cells.

### Expression of chemokine/cytokine encoding genes by bursal M cells

**Figure 5:**
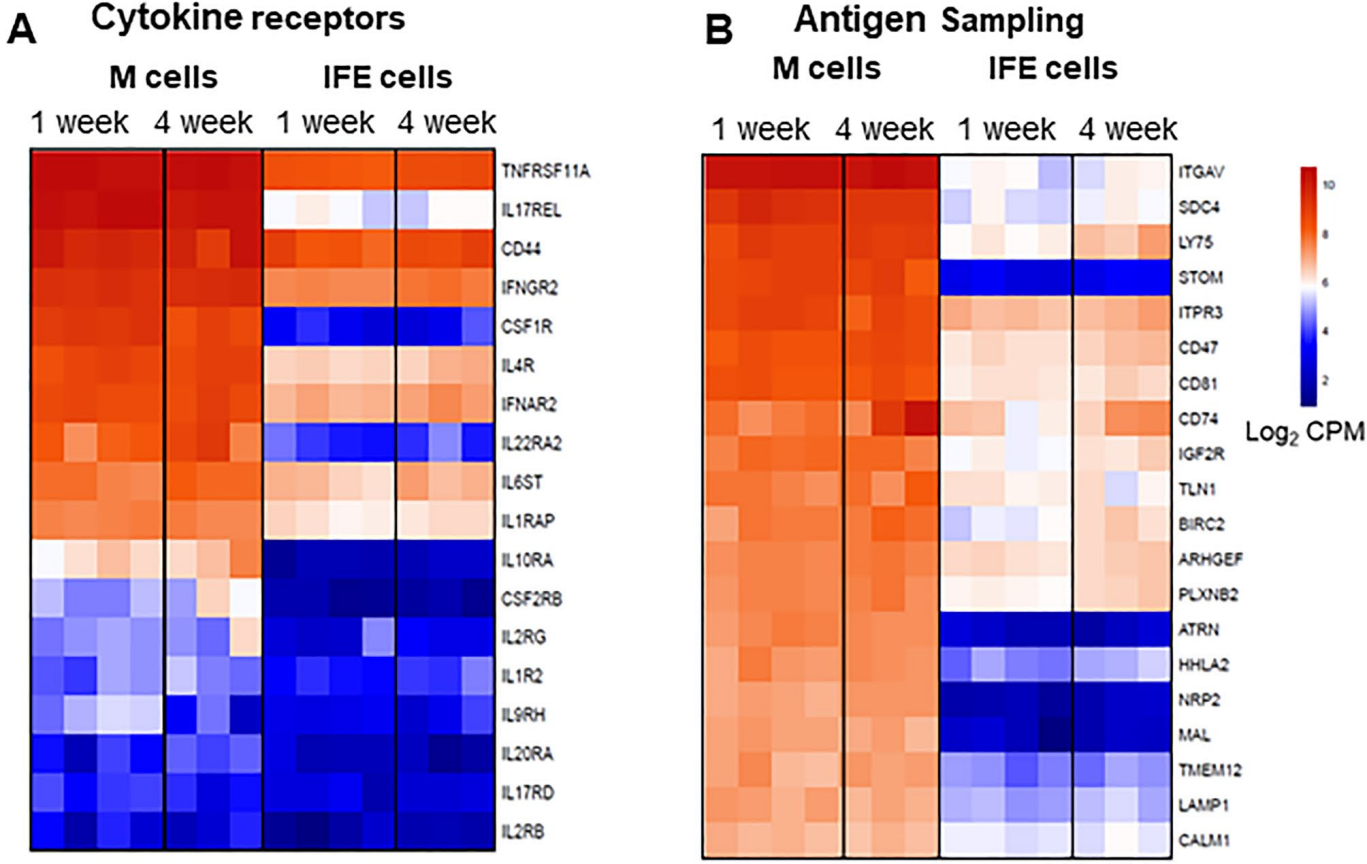
Identification of cytokine signalling receptors and putative functional molecules associated with bursal M cells. (**A**) Top 20 upregulated genes in sorted bursal M cells and IFE cells from 1-week and 4-week-old chickens associated with (**A**) cytokine receptor signalling, (**B**) endocytic, G-protein-coupled, and surface receptors using gProfiler and UniProt. Genes were identified based between 1-week-old M cells and IFE cells. Data represent n = 4 for 1-week and n = 3 for 4-week-old chickens. Heatmaps colouring shows the relative expression level log_2_ CPM (counts per million) of genes (rows) across cells (columns) with red indicating high counts and blue indicating low counts.

To further identify candidate genes involved in M cell development and function, our list of 2260 genes were filtered using the ‘cytokine-mediated signalling pathway’ GO term (GO:0019221) and ‘transmembrane signal receptor’ Panther protein classification (Supplementary File 5). In mouse, M cell differentiation is critically dependent on stimulation from RANKL produced by subepithelial stromal cells beneath the FAE^10^. This cytokine signals via its receptor, RANK, which is expressed throughout the gut epithelium^10^. Here, higher levels of the expression of *TNFRSF11A* (encoding RANK) were detected in M cells compared to IFE (Fig. 5A), consistent with studies in mice^9^. In addition to *CSF1R*, we also found an enrichment of interleukin (IL) receptor genes in bursal M cells, such as *IL17REL*, *IL4R*, *IL22RA2*, *and IL10RA* at both ages (Fig. 5A). The chemokine genes, *Ccl9*, *Ccl20* and *Cxcl11* are highly expressed in the FAE of mice, working to recruit leukocytes. None of these genes were upregulated in chicken M cells (Supplementary File 1). The most highly expressed chemokine in our dataset was *CCL19* (Supplementary File 2) which may function to attract CCR7 expressing macrophages and DC^44^. Collectively, these data imply that chicken M cells express a distinct array of cytokine receptors, suggesting a potential role of their signalling in the development or function of M cells.

### Chicken bursal M cells are enriched with genes associated with antigen sampling

M cells actively transport luminal antigens across the epithelial barrier via transepithelial pathway known as transcytosis. Functional enrichment (GO terms) and KEGG signalling pathways analysis were performed on the upregulated gene dataset using gProfiler (Supplementary File 4). Murine M cells express GP2, UMOD, and PRNP on their apical surface to transport the luminal antigens across the epithelial barrier^15,21,32^. Whereas PRNP was enriched in chicken M cells implying a similar role in the uptake of certain microorganisms or antigens into these cells, UMOD was not present and the gene encoding GP2 is not annotated in the chicken genome. To identify potential genes associated with transcytosis by chicken M cells, we filtered the upregulated genes of bursal M cells for endocytic, G-protein-coupled, and surface receptors genes using gProfiler and UniProt. A full list of 376 genes is provided in Supplementary File 6 and the top 20 genes are shown in Fig. 5B. The genes were largely categorised in following groups; transmembrane signal receptor (*FGFR1*, *IGF1R*, *PLXNB2*, *SDC4*), scaffold/adaptor protein (*CD151*, *CD63*, *CD81*, *TSPAN4*), membrane traffic protein (*CAV2*, *DNM1*, *LAMP1-2*, *LY75*, *PLA2R1*), integrin (*ITGA6*, *ITGAV*, *ITGB2*, *ITGB8*), G-protein (*ADIPOR2*, *GNAI2*, *GNB1*, *GNB4*), non-motor actin binding protein (*CTNNA1*, *CTNNB1*, *CTTN*), and transporter (*ATP11C*, *ATP2B4*, *ATP6V1H*, *SLC9A3*). *ITGAV* was highly upregulated in M cells compared to IFE cells. ITGAV forms a heterodimer with ITGB8, which also found highly expressed in chicken M cells (Fig. 3A). *LY75* (DEC-205 or CD205) is a C-type lectin found to be enriched in chicken M cells. LY75 is expressed on murine DC, B and T cells, and thymic epithelial cells and has endocytic capacity^48,49^. In addition, genes involved in ion channel regulation (*STOM*)^50^, immune cell attraction (*ATRN*)^51^, vesicular trafficking (*MAL*)^52^, and transmembrane glycoprotein, *NRP2* were expressed at substantially higher levels in M cells compared to IFE cells and may indicate their role in subcellar transport of molecules in M cells. In contrast to murine M cells, which express lower levels of LAMP1 compared to FAE enterocytes^53^, *LAMP1* was highly expressed in chicken bursal M cells compared to IFE cells which was also observed at a protein level^24^.

Members of Annexin family have been implicated in antigen transportation and cell morphology^54^. Studies suggest that ANXA5 in murine M cells is associated with cytoskeletal regulation and endocytic pathways^11,43^. ANXA4 and ANXA11 induce membrane curvature and promote vesicle aggregation^55–57^, and are highly enriched in chicken M cells (Fig. 3A). Therefore, it is likely that ANXA4 and ANXA11 play analogous roles in chicken M cells. Similarly, AIF1 is involved in particulate uptake in murine M cells^36^. However, it is not present in current chicken genome and instead AIF1L, a gene that shares 60% homology with AIF1, was found to be highly enriched in bursal M cells. Together these data suggest chicken M cells express many genes associated with antigen-uptake and subcellular transport.

### M cells in the chicken small intestine lack CSF1R expression

**Figure 6:**
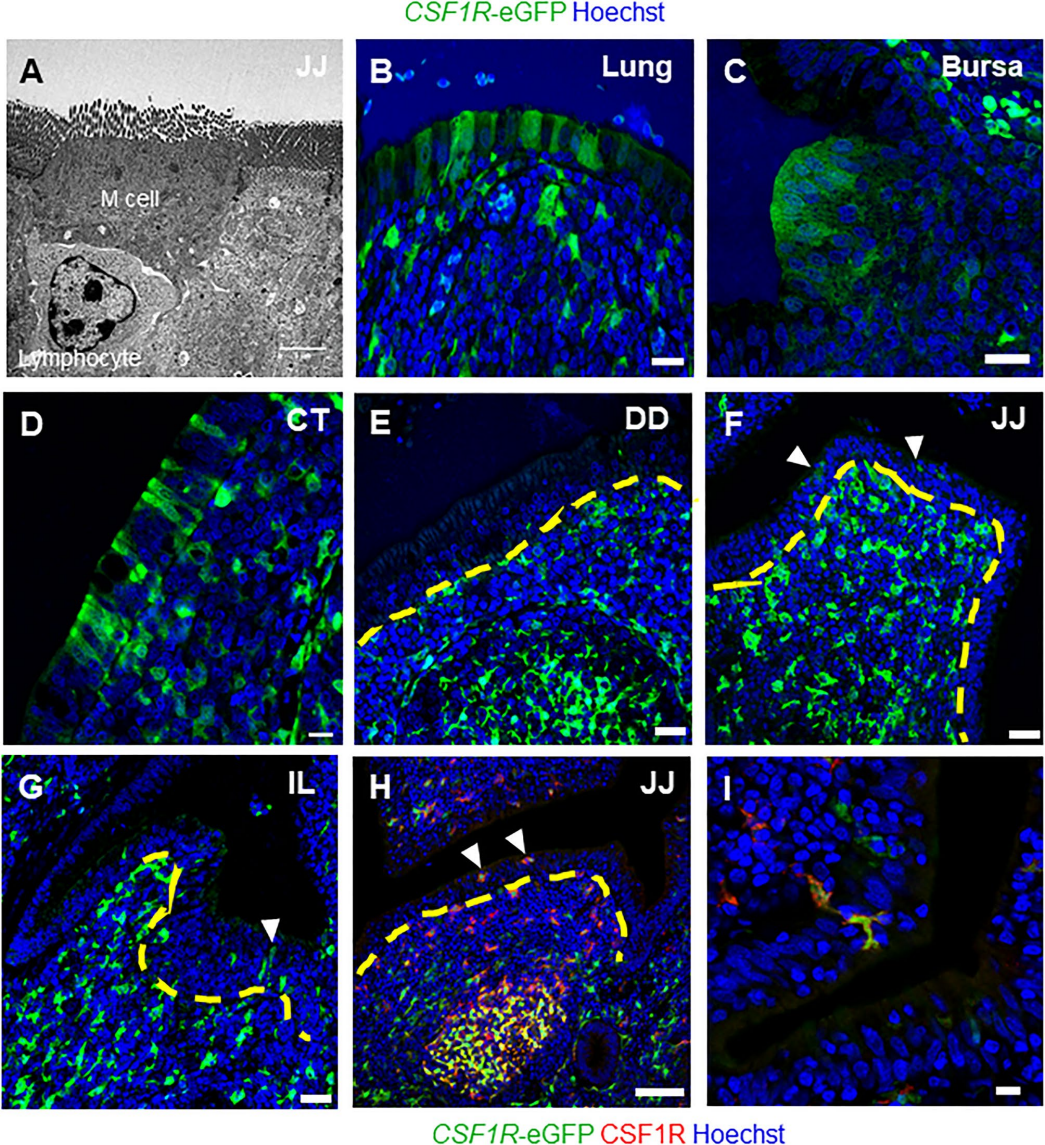
FAE of the small intestine lack *CSF1R-*eGFP and CSF1R expression. (**A**) TEM analysis of the jejunal PP reveals the presence of M cells with the characteristic horse-shoe shape morphology and intraepithelial pocket harbouring a lymphocyte. Immunofluorescent analysis of the FAE in the (**B**) lung, (**C**) bursa, and (**D**) caecal tonsil (CT) demonstrates the cuboidal morphology, characteristic of an epithelial cell, of *CSF1R*-eGFP^POS^ M cells. Immunofluorescent analysis of the FAE in the PP regions of (**E**) the duodenum (DD), (**F**) jejunum (JJ) and (**G**) ileum (IL) demonstrates the lack of *CSF1R*-eGFP^POS^ epithelial cells (yellow dashed line). *CSF1R*-eGFP^POS^ macrophages and DC (white arrows) can be observed near the epithelial cell layer. Scale bars = 50 µm. (**H**) FAE cells of the jejunal PP do not express CSF1R protein (red), demonstrating lack of *CSF1R*-eGFP (green) in small intestinal M cells is not due to the promoter regions used for the development of the CSF1R transgenic chickens. (**I**) The *CSF1R*-eGFP^POS^ CSF1R^POS^ cells within the epithelial cell layer represent extended dendrites of mononuclear phagocytes and not M cells. Scale bars = 100 and 50 µm. Data is representative of four independent experiments using 4-week-old *CSF1R*-eGFP reporter transgenic chickens.

Transmission electron microscopical analysis on the FAE overlying the PP in the jejunum confirmed the existence of M cells exhibiting horseshoe-shaped morphology and an intraepithelial pocket harbouring immune cells (Fig. 6A). Our studies using *CSF1R*-reporter chickens have shown that M cells within the FAE of the BALT^23^, bursa, caecal tonsils (Fig. 6B-D), and proctodaeum^24^ express *CSF1R*^23,24^. However, whether M cells in the FAE of the chicken small intestine similarly express *CSF1R* was unknown. We therefore analysed the expression *CSF1R*-eGFP in the PP regions of the duodenum, jejunum, and ileum of 4-week-old *CSF1R*-reporter transgenic chickens (Fig. 6E–G). In contrast to the bursa, expression of *CSF1R*-eGFP^POS^ cells and CSF1R protein was undetectable in the FAE throughout the small intestine (Fig. 6H). Although occasional *CSF1R*-eGFP^POS^ cells were detected in the epithelial layer of the jejunum and ileum, these cells lacked the characteristic epithelial cell shape and may represent intra-epithelial cells. As anticipated, the expression of CSF1R protein was detected in macrophages/DC in the underlying lamina propria (Fig. 6H-I). This analysis also supported the idea that the *CSF1R*-eGFP^POS^ cells detected within the epithelial layer were *filopodia* protruding through the epithelial layer (Fig. 6I, Supplementary Video 1). Together, these data show that in contrast to the lung, large intestine, and the bursa, M cells in the FAE of the small intestine do not express CSF1R.

### SOX8 as common M cell gene marker

**Figure 7:**
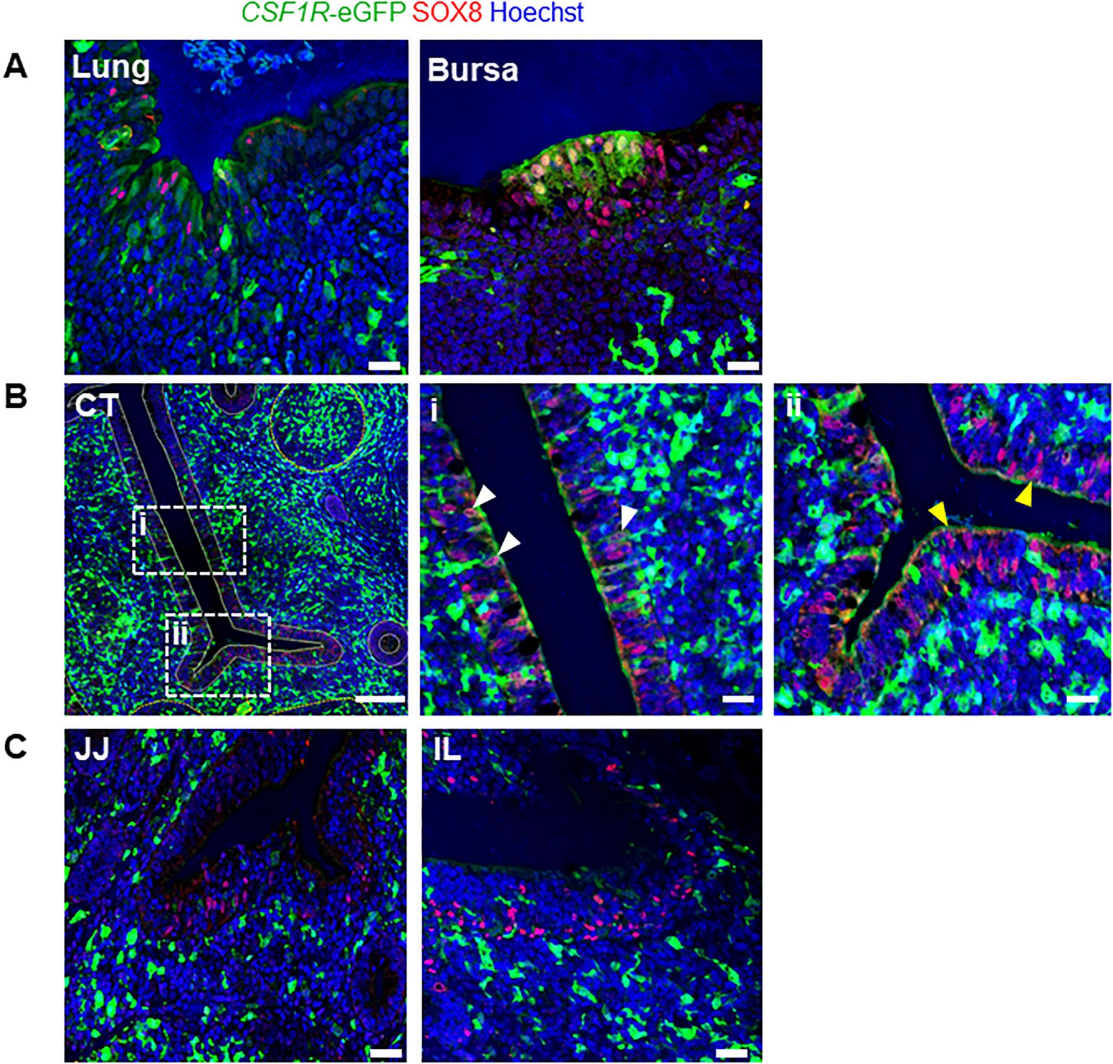
Chickens exhibit diversity in their M cells characterised by CSF1R expression. (**A**) SOX8 expression co-localises with the *CSF1R*-eGFP^POS^ FAE cells in the BALT and the bursa. (**B**) *CSF1R-*eGFP is differentially expressed in SOX8^POS^ M cells in the caeca. (**Bi**) *CSF1R-*eGFP^*POS*^ SOX8^POS^ cells are evident in the higher regions of the villi. (**Bii**) *CSF1R*-eGFP^NEG^ SOX8^POS^ cells are predominantly located in the crypt regions. (**C**) SOX8 expression in the FAE regions of the jejunum (JJ) and ileum (IL) indicating diversity in chicken M cells associated with CSF1R expression. Data is representative of three independent experiments using 4-week-old *CSF1R*-eGFP reporter transgenic chickens. Scale bars = 100 and 10 µm.

Finally, we determine whether the CSF1R^NEG^ M cells in the small intestine expressed the key transcriptional regulator SOX8 (Fig. 7). Consistent with data from the bursa (Fig. 3A), SOX8 expression was detected in epithelial cells in the BALT and small intestine (Supplementary Fig. 1). Immunofluorescent analysis identified strong SOX8 expression in *CSF1R*-eGFP^POS^ M cells in the lung and bursa (Fig. 7A). In caecal tonsils, both SOX8^POS^ *CSF1R*-eGFP^POS^ and SOX8^POS^ *CSF1R*-eGFP^NEG^ cells were observed in the epithelium (Fig. 7Bi–ii), with the latter being confined to the lower crypt regions. In the jejunum and ileum, SOX8 staining was observed in *CSF1R*-eGFP^NEG^ cells within the FAE regions (Fig. 7C). Together, these data reveal that whereas the M cells in the BALT, bursa and caecal tonsils and small intestine of chickens all express SOX8, two subpopulations were specifically identified in the small intestine that differed in their expression of CSF1R.

## Discussion

Here, we utilised *CSF1R*-eGFP reporter transgenic chickens to specifically enrich *CSF1R*-expressing bursal M cells and analysed their transcriptome using mRNA-seq. Our analysis identified genes conserved between mouse and chicken M cells while also identifying genes specific to chicken bursal M cells. In turn, we demonstrate that chickens may have two subsets of M cells in the small intestine based on their expression of CSF1R.

In mammals, the regulation of M cell development is orchestrated by the RANK-RANKL signalling and intrinsic expression of the transcription factors Spi-B and SOX8, leading to the differentiation of functionally mature GP2^POS^ M cells^13,32^. There are no current annotations for the genes that encode SPIB and GP2 in the chicken genome. Chicken bursal M cells express CSF1R highly, suggesting their ontogeny and/or developmental pathway may differ from mammalian M cells which do not express CSF1R^22^. Through transcriptome analysis we identified 2260 DEGs which were specifically upregulated in chicken M cells compared to IFE cells. Within this dataset elevated transcript levels of murine M-cell-associated genes, *MARCKSL1*, *NFKB2*, *PRNP*, *SOX8*, *TNFAIP2*, *RELB*, *TNFRSF11B*, and *TNFRSF11A*, were present suggesting the conservation of mammalian M cell developmental or functional pathways in chickens^12,14,21,31,37^. Moreover, chicken M cells expressed a significant abundance of many gene transcripts from closely related mouse M cell-associated gene families of the absent genes, notably those implicated in lineage determination, for example *SPI1*, and essential cellular functions as discussed below.

Cell differentiation in the intestinal epithelium is governed by transcription factors, some of which exhibit highly lineage-restricted expression patterns. The induction of M cells relies on the SOX8 and Spi-B transcription factors^13,14,32^. In this study, *SOX8* and a paralog of *SPIB*, *SPI1*, were found to be highly upregulated in bursal M cells compared to IFE cells. Indeed, our analysis suggested that 450 of the M cell-associated DEGs contained SPI1 binding motifs in their promoter regions^58,59^. In mice, *Spi1* has the capacity to compensate for the loss of *SPIB*, and this compensatory mechanism may suggest a similar role for *SPI1* in the functional differentiation of chicken M cells^60^. The origin of bursal FAE cells is currently uncertain with evidence supporting their mesenchymal and haematopoietic origins^24,61–63^. The expression of PU.1 (encoded by SPI1) is consistent throughout the hematopoietic hierarchy and orchestrates the differentiation of myeloid cells in concert with CEBPA^64^. In mammals, cell differentiation beyond the pre-granulocyte–macrophage (GM)/common myeloid progenitor stage is blocked in the absence of CEBPA, enabling the cells to follow a non-GM lineage developmental pathway^65^. We found no enrichment of *CEBPA* or *CEBPB* gene transcripts in chicken M cells but did observe elevated transcript levels of genes associated with haematopoiesis, such as *CSF1R*, *HIF1A*, and *STAT3*^66–68^. Although the elevated transcript levels of *SPI1* and *CSF1R* would suggest chicken M cells have a similar transcriptional profile as macrophages, however, no enrichment of macrophage gene signatures ^69,70^ suggest that bursal M cells like mammalian M cell do not follow a similar transcriptional pathway as macrophages.

Although bursal M cells are efficient at the uptake of 0.02–0.1 µm latex beads, they were impaired in the uptake of larger particles and bacteria, such as *Salmonella* bacteria^24^. Few known transcripts of surface receptors related to endocytosis activity and conserved pathogen capture receptors were upregulated in bursal M cells. Those that were detected included *LY75*, *NRP2*, *CD44*, *CD151*, *CD81* and the conserved murine M cell gene, *PRNP* ^21,71–75^. Genes associated with actin remodelling processes, including *AIF1L*, *ANAX4*, *ANAX11*, *CD63*, exhibited significant expression levels in bursal M cells, suggesting their capability to modify cellular structure for particle uptake^76^. The most upregulated integrins in bursal M cells were *ITGB8* and *ITGAV*, which form the αvβ8 complex. This strictly expressed complex mediates TGFβ activation in murine lung epithelial cells leading to the recruitment of immune cells^77^. Along with its ability to control TGFβ activation, αvβ8 may play a role in regulating immune-M cell interaction. A number of chemokines transcripts were elevated in bursal M cells compared to IFE cells. Murine M cells express CCL9 and CCL20 which attract B cells and DC towards the M cells^78^, but both genes were not present in our dataset. Both CCL20 and Psg18 expression in the FAE of mouse are dependent on RANK-TRAF6 mediated NF-κB signalling^30^. We observed no upregulation of TRAF6 in chicken M cells which may explain the lack enrichment of these genes. We did find CCL19 enriched in chicken M cells which may attract CCR7^POS^ DC^44^. In addition, low transcript levels of *CCL26* were present in chicken M cells. In mice the expression of *CCL26* in M cells mediates the recruitment of CX3CR1^POS^ DC^79^. Chicken bursal M cells exhibited a notable enrichment of receptors that regulate the bioactivity of cytokines and inflammation, such as *IL17REL, IL22RA2*, *IL1R2*, *IL1RAP*, and *IL10RA*^80^. In mice, the negative regulation of IL-22 signalling in the FAE by IL-22-binding protein-expressing DC is important for facilitating the transcytosis of antigens in PP^81^. In addition, bursal M cells also exhibited moderate transcripts for *IFNGR2* and *IFNAR2*, indicating their ability to respond to IFNα/γ supported by their enrichment of transcriptional regulators of anti-viral responses, *ELF1* and *ELF5*^82,83^. Gut-derived molecules, are critical for post-hatch B cell development and believed to be transported through the bursal M cells^84^. Thus, the restricted expression of endocytic or pathogen capture receptors on bursal M cells may have a role in controlling entry of gut-derived particulates to regulate the proliferation and maturation of B cells within the underlying follicles.

**Figure 8:**
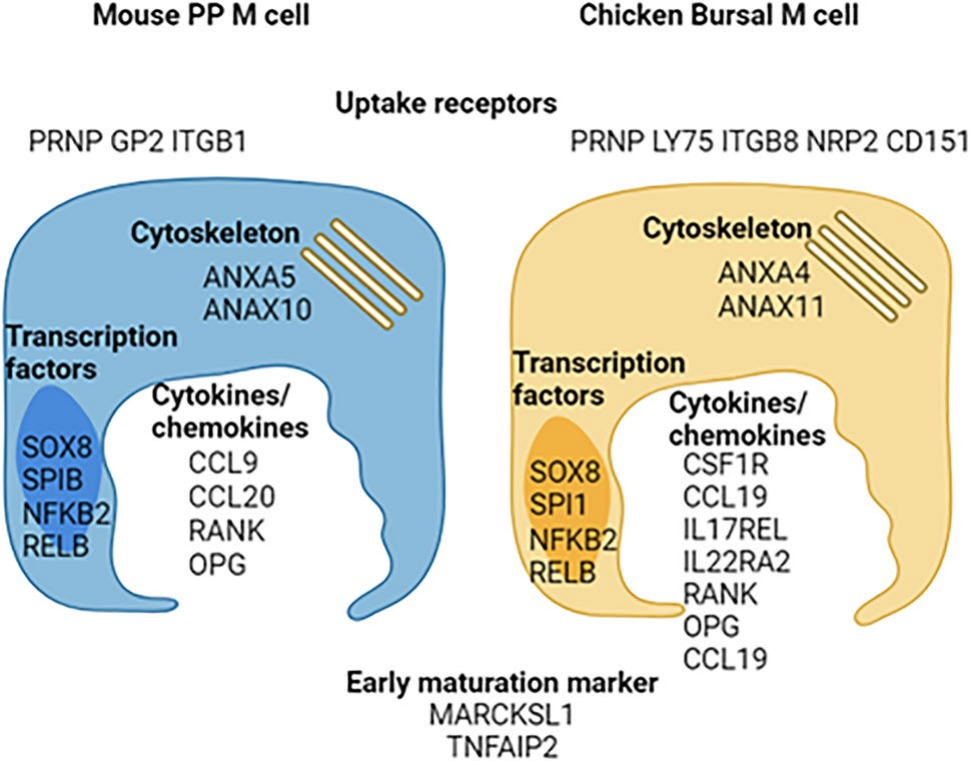
Schematic overview of M cell-associated genes in mouse PP M cell and chicken bursal M cell. Image created using Biorender (www.biorender.com).

The histological examination of the FAE cells throughout the intestine revealed that small intestinal M cells lacked expression of CSF1R mRNA and protein, and while SOX8 is highly conserved in all chicken M cells, the expression of CSF1R is tissue-specific. This implies that there are key differences the transcriptional programs between these two types of M cells. Further work is required to determine the relationship between chicken *CSF1R*^POS^ SOX8^POS^ and *CSF1R*^NEG^ SOX8^POS^ M cell lineages. A schematic overview of the conserved and unique genes identified in chicken bursal M cells is provided in Fig. 8.

In summary, the gene expression profile of *CSF1R*^POS^ bursal M cells demonstrates the conservation of murine M-cell associated genes but also chicken-specific gene signatures. In addition, we have identified diversity between chicken M cells located in the small and large intestine based on CSF1R expression. Future studies will resolve the developmental difference between CSF1R^POS^ and CSF1R^NEG^ M cells and in defining the functionality of M cells in the small intestine to help improve mucosal immune responses through vaccine targeting.

## Methods

### Experimental animals and ethics statement

*CSF1R*-eGFP reporter transgenic and non-transgenic, Hy-line Brown, chickens were provided by the National Avian Research Facility, The Roslin Institute, University of Edinburgh, UK. Birds received food and water ad libitum and were not vaccinated. Breeding of transgenic chickens was carried out under the authority of Project License PPL 70/8940 with the consent of The Roslin Institute Animal Welfare and Ethical Review Board and experiments were carried out in accordance with the UK Home Office project licence (PP8571233) and in compliance with the UK Home Office Animals Act 1986 (Scientific Procedures), and these ensured they were carried out in compliance with the ARRIVE guidelines and recommendations.

### Immunohistology

The bursa, PP from the duodenum, jejunum and ileum, caecal tonsils, and lung of *CSF1R*-eGFP reporter transgenic and non-transgenic chickens were collected and fixed in 10% neutral buffered formalin (NBF, Cell Path) for 24 h at RT and embedded in paraffin. Tissues were sectioned at 8 µm thickness and mounted on Superfrost Plus slides (Thermo Fisher Scientific (TFS)) from a 42 °C water bath and incubated at 50 °C overnight. For IHC, formalin-fixed paraffin-embedded (FFPE) sections were dewaxed and antigen retrieval was performed for SOX8 and AIF1L antibodies in 10 mM sodium citrate (Sigma-Aldrich) buffer at 121 °C for 15 min. Slides were washed in staining buffer (PBS, 0.1% BSA and 0.01% Tween-20, Sigma) for 5 min at RT and incubated with 5% horse (Sigma) or goat serum (Chondrex) for 1 h. Sections were stained with primary antibodies (Supplementary Table 3) for 3 h at RT. For biotin-conjugated antibodies, ABC solution (Vector Laboratories) was applied to the sections for 30 min. Sections were visualised using diaminobenzidine (DAB; Sigma-Aldrich), counterstained with Haematoxylin (Sigma-Aldrich) and mounted using Pertex mounting medium (Cell Path). For immunofluorescence staining, after incubation with primary antibodies, sections were incubated with fluorophore-conjugated secondary antibodies and Hoechst 33258 (Sigma-Aldrich) for 1 h at RT. Slides were mounted using Prolong Diamond Anti-fade mountant (Invitrogen). Brightfield and fluorescence images were captured using Nikon light microscope and Zeiss LSM 880 confocal microscope, respectively. Images were analysed using ZEN software (Carl Zeiss).

### Transmission electron microscopy (TEM)

PP regions from the jejunum of 4-week-old chickens were isolated based on *CSF1R*-eGFP expression and fixed in 3% glutaraldehyde in 0.1 M sodium cacodylate buffer, pH 7.3, for 2 h and processed as described previously^85^. Ultrathin sections (60 nm) were stained in uranyl acetate and lead citrate and imaged using a JEOL JEM-1400 Plus TEM and analysed using ImageJ v1.52e (Fiji).

### Isolation of bursal cells

Isolation of bursal epithelium was performed as previously described^21^. In brief, bursas were removed and stored in ice-cold PBS until use. Bursas were opened to reveal the epithelial surface and digested in 50 mM EDTA (in-house) in Mg^2+^Ca^2+^ free Hanks’ balanced salt solution (HBSS, Corning) for 30 min at 400* g* at 37 °C. The supernatant was collected, filtered through a 70 µm sieve and centrifuged at 400 g at 4 °C for 10 min. The number of live cells was determined by Trypan blue (Corning) exclusion using a Haemocytometer and the cell number was adjusted to 10^7^ cells/mL for flow cytometric analysis.

### Flow cytometry

One million isolated bursal cells were stained with primary and secondary antibodies, outlined in Supplementary Table 3, on a 96-well U-bottom plate (TFS), on ice, in the dark for 30 min. Cells were pelleted at 190 g for 3 min and washed twice with FACS buffer (PBS, 0.5% BSA and 0.001% sodium azide, Sigma). Antibodies were diluted in FACS buffer. Compensation was achieved using BD™ CompBeads anti-mouse Igκ particles (BD Bioscience) for each flurochrome. Fluorescence minus one (FMO) and secondary, isotype controls were prepared as described previously^69^. Phenotypical analysis was performed on the LSRFortessa™ with 4 lasers and 16 filters (BD Biosciences). Sytox™ Blue Dead Cell Stain (TFS) was added 5 min prior to flow analysis to differentiate dead and live cells. Cell gating was carried out using cells from non-transgenic chickens and FMO controls. At least 10,000 to 100,000 live, single cell events were captured for analysis. Data were analysed using the software program FlowJo (Tree Star v10).

### Fluorescence-activated cell sorting (FACS)

To enrich for bursal M cells and IFE cells for RNA-seq, 3 bursas from 1-week-old birds and two bursas from 4-week-old birds were pooled for each biological replicate (1-week-old, n = 4 biological replicates and 4-weeks-old, n = 3 biological replicates). For each sorting experiment, an age-matched, non-transgenic chicken was used to apply cell gates. Bursal segments were digested in 50 mM EDTA in HBSS containing RNasin® Plus RNase inhibitor (1:100, Promega) as outlined above. Cells were stained with mouse anti-chicken CD45-APC as described in the previous section. Cell numbers were adjusted to 8 × 10^7^ cells/tube in 500 µL of FACS buffer supplemented with RNase inhibitor. Prior to the cell sort, Sytox™ Blue Dead Cell Stain (TFS 1:1000) was added to the sample. Cells were sorted using FACS ARIA™ III cell sorter, with 4 lasers and 11 detectors (BD Biosciences). CD45^NEG^ *CSF1R*-eGFP^POS^ (M cells) and CD45^NEG^ *CSF1R*-eGFP^NEG^ cells (IFE) were collected into ice-cold 1.5 mL tubes (TFS), containing 200 µL of FACS buffer supplemented with RNase inhibitor.

### RNA extraction

Sorted bursal M cells and IFE cells were pelleted at 8000 g at 4 °C for 10 min immediately after sorting and lysed with RLT Plus buffer (Qiagen) supplemented with 2-Mercaptoethanol (2-ME, Sigma-Aldrich) and stored at  − 80 °C until use. Total RNA was purified using RNeasy Plus Micro Kit (Qiagen) with a genomic-DNA eliminator column according to the manufacturer’s instructions. The quality and integrity of the RNA was analysed using high sensitivity RNA ScreenTape Kit (Agilent) according to the manufacturer’s instructions. Samples were analysed on the Agilent 2200 TapeStation instrument (Agilent) and samples with RNA integrity number (RIN) of 9 or more were selected for RNA sequencing.

### RNA sequencing and data analysis

Reads were trimmed for quality at the 3’ end with a threshold of 30 and for adapter sequences using Cutadapt [version cutadapt-1.9.dev2^86^]. A minimum length of 50 was retained after trimming. Reads were aligned to *Gallus gallus* genome (GRCg6a) from Ensembl using STAR [version 2.5.2b^87^]. Genes with near-zero counts were filtered based on counts per million (CPM), where a row of the expression matrix was required to have values greater than 0.1 in at least four samples. Reads were normalised using the weighted trimmed mean of M-values (TMM) method^88^. Differential gene expression analysis and principal component (PC) analysis was carried out using edgeR (version 3.36.0), where maximum false discovery rate (FDR) < 0.05 and minimum Log_2_ fold change (FC) > 1 for upregulated DEGs and Log_2_ FC < − 1 for downregulated DEGs. Statistical assessment of differential expression was analysed using quasi-likelihood F-test. Functional enrichment analyses including Gene Ontology (GO) and Kyoto encyclopedia of genes and genomes (KEGG) pathway were performed using gProfiler. Transcription factors, cytokine receptors and cell surface receptors were obtained from the UniProt database and AmiGO database and categorised using Panther Classification (System v.14.0).

### Ethics statement

All procedures were conducted under Home Office project license PE263A4FA according to the requirements of the Animal (Scientific Procedures) Act 1986, with the approval of The Roslin Institute’s Animal Welfare and Ethical Review Board. Embryos were humanely culled in accordance with Schedule 1 of the Animals (Scientific Procedures) Act 1986.

### Supplementary Information

Supplementary Information 1.

Supplementary Information 2.

## Data Availability

Sequence data that support the findings of this study have been deposited in the European Nucleotide Archive (ENA) at EMBL-EBI under accession number PRJEB61278.
