# Supplementary material for: Discrimination of distinct chicken M cell subsets based on CSF1R expression
Source: Sci Rep. 2024 Apr 16;14:8795. doi: 10.1038/s41598-024-59368-x (PMC11021470; doi:10.1038/s41598-024-59368-x)
Supplement: Supplementary file 1 — Supplementary Information 1. [file 41598_2024_59368_MOESM1_ESM.pdf]

Discrimination of distinct chicken M cell subsets based on CSF1R expression

Safieh Zeinali, Kate Sutton, Masoud Ghaderi Zefreh, Neil Mabbott, Lonneke Vervelde

Supplementary material:

Supplementary Table 1 Assessing the transcriptome of sorted CSF1R-transgene^{POS} bursal M cells for the presence (green) and absence (red) of upregulated genes identified as conserved between murine PP- and chicken bursal-enriched M cells in Nakato et al., (2009) microarray study. Blue cells indicate the genes which are downregulated in bursal M cells. Asterisks show *CUGBP2* and *SACDS* whose names have been changed to *CELF2* and *SACS*, respectively.

Supplementary Table 2. Assessing the upregulated DEG of sorted *CSF1R*-transgene^{POS} bursal M cells for the presence (green) and absence (red) of upregulated genes in murine PP M cells (Haber et al., 2017). Blue cells indicate the genes which are downregulated in bursal M cells. Asterisks show *1700011H14Rik* and *BC021614* whose names have been changed to *Ccdc198* and *Gstp3*, respectively.

Supplementary Table 3. List of primary and secondary antibodies and isotype controls used for flow cytometry and immunostaining.

Supplementary Video 1. Z stack image of *CSF1R*-transgene^{POS} CSF1R^{POS} mononuclear phagocyte (white arrow) protruding dendrites through the epithelial layer in the jejunum PP.

Supplementary Figure 1. Expression of SOX8 in M cells of chicken lung and small intestine. FFPE sections from lung, caecal tonsil (CT), and PP of the duodenum (DD), jejunum (JJ), and ileum (IL) were stained with rabbit anti-human SOX8 (brown nuclei) and nuclei were counterstained with haematoxylin. Data are representative of 3 biological replicates for duodenal, jejunal, and ileal PP and CT birds and 2 biological replicates for the BALT from 7-week-old *CSF1R*-reporter transgenic chicken.

Supplementary Files 1-6. List of the upregulated genes in bursal M cells and genes filtered based on GO and gProlifer.

Supplementary Table 1. Assessing the transcriptome of sorted *CSF1R*-transgene^{POS} bursal M cells for the presence (green) and absence (red) of upregulated genes identified as conserved between murine PP- and chicken bursal-enriched M cells microarray study[21]. Blue cells indicate the genes which are downregulated in bursal M cells. Asterisks show *CUGBP2* and *SACDS* whose names have been changed to *CELF2* and *SACS*.

ACTN1		*POLL*	
ANXA10		*POU2AF1*	
BUB1		*PRNP*	
CD44		*RAC2*	
CLU		*SACDS**	
*CUGBP2**		*SAMSN1*	
CXCR4		*SGSH*	
EVL		*SLBP*	
FABP5		*SLC16A3*	
FKBP1A		*TNFRSF1B*	
FLI1		*TNFRSF19*	
GLIPR1		*TRAF3*	
GPR137B		*TYRO3*	
LCP1		*VIM*	

Supplementary Table 2. Assessing the upregulated DEG of sorted *CSF1R*-transgene^{POS} bursal M cells for the presence (green) and absence (red) of upregulated genes in murine PP M cells single cell RNA-seq study[45]. Blue cells indicate the genes which are downregulated in bursal M cells. Asterisks show *1700011H14Rik* and *BC021614* whose names have been changed to *Ccdc198* and *Gstp3*, respectively.

Ccl20	red	*Adgrd1*	red
Clu	red	*Ncf4*	green
Mfge8	red	*Rnf128*	green
Anxa5	red	*Il4i1*	green
Pglyrp1	red	*Far2*	red
Ctsh	red	*BC021614**	red
Serpinb6a	red	*D630011A20Rik*	red
H2-M2	red	*Vcam1*	red
Gp2	red	*Stx11*	red
Ubd	red	*Sdhaf1*	red
Lamp1	green	*Ces1b*	red
Cxcl16	red	*Itga3*	red
Cyba	green	*Msln*	blue
Scd1	red	*Scarb2*	green
*1700011H14Rik**	red	*Tnfrsf4*	red
Aif1	red	*Fam98a*	red
Ctsd	red	*Tmsb4x*	green
Tnfaip2	green	*Nfkbia*	red
Far2os2	red	*Rnase1*	red
Slc2a6	green	*Vamp5*	red
Gulo	red		

Supplementary Table 3. List of primary and secondary antibodies and isotype controls used for flow cytometry and immunostaining.

Antibody (clone), isotype	Antigen	Product info.	Conc.
Mouse anti-chicken MHCII (2G11), IgG1κ PE	MHC II	Southern Biotech 8350-09	1 µg/mL
Mouse anti-chicken CD45 (LT40), IgM APC	CD45	Southern Biotech 827011	1 µg/mL
Mouse anti-chicken KUL01, IgG1 PE	MRC1L-B	Southern Biotech 8420-09	1 µg/mL
Mouse anti-chicken CD11c (8F2), IgG1	Putative CD11c	Gifted by S.Härtle, LMU (Germany)	5 µg/mL
Mouse anti-chicken CD44 (AV6) IgG1 (s/n)	CD44	Institute for Animal Health (UK)	1:500
Mouse anti-chicken TIM4 (JH9), IgG1	TIM4	Immunological Toolbox, The Roslin Institute (UK)	2 µg/mL
Mouse anti-chicken CSF1R (AV170), IgG1	CSF1R		2 µg/mL
Rabbit anti-human SOX8, IgG (polyclonal)	SOX8	Abcam ab221053	2 µg/mL
Rabbit anti-human AIF1L, IgG (polyclonal)	AIF1L	Abcam ab204493	0.25 µg/mL
Mouse anti-GFP (7.1 & 13.1), IgG1κ	GFP	Roche 11814460001	2 µg/mL
Rabbit poly anti-GFP AF488®	GFP	ThermoFischer	2 µg/mL
Mouse Isotype IgG1κ-PE		BioLegend 400112	Various
Rabbit Isotype IgG		Abcam ab37415	Various
Goat anti-mouse IgG1 AF568®	Mouse IgG1	Invitrogen A21124	2 µg/mL
Goat anti-mouse IgG AF568®	Mouse IgG	Southern Biotech A-11031	4 µg/mL
Goat anti-rabbit IgG AF594®	Rabbit IgG	Abcam ab150080	4 µg/mL
Goat anti-rabbit IgG Biotin	Rabbit IgG	Southern Biotech 4010-08	1 µg/mL

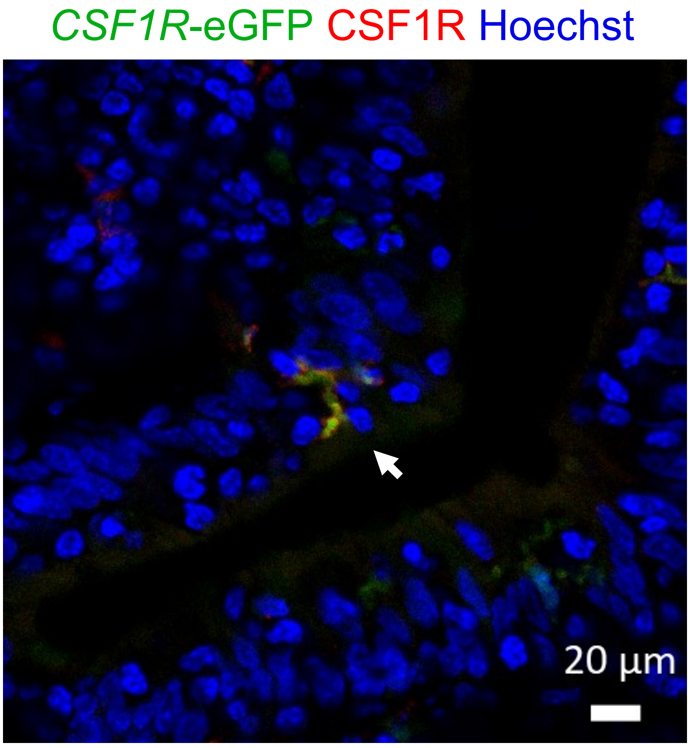

Supplementary Video 1. Z stack image of *CSF1R*-transgene^{POS} CSF1R^{POS} mononuclear phagocyte (white arrow) protruding filopodia through the epithelial layer in the jejunum PP.

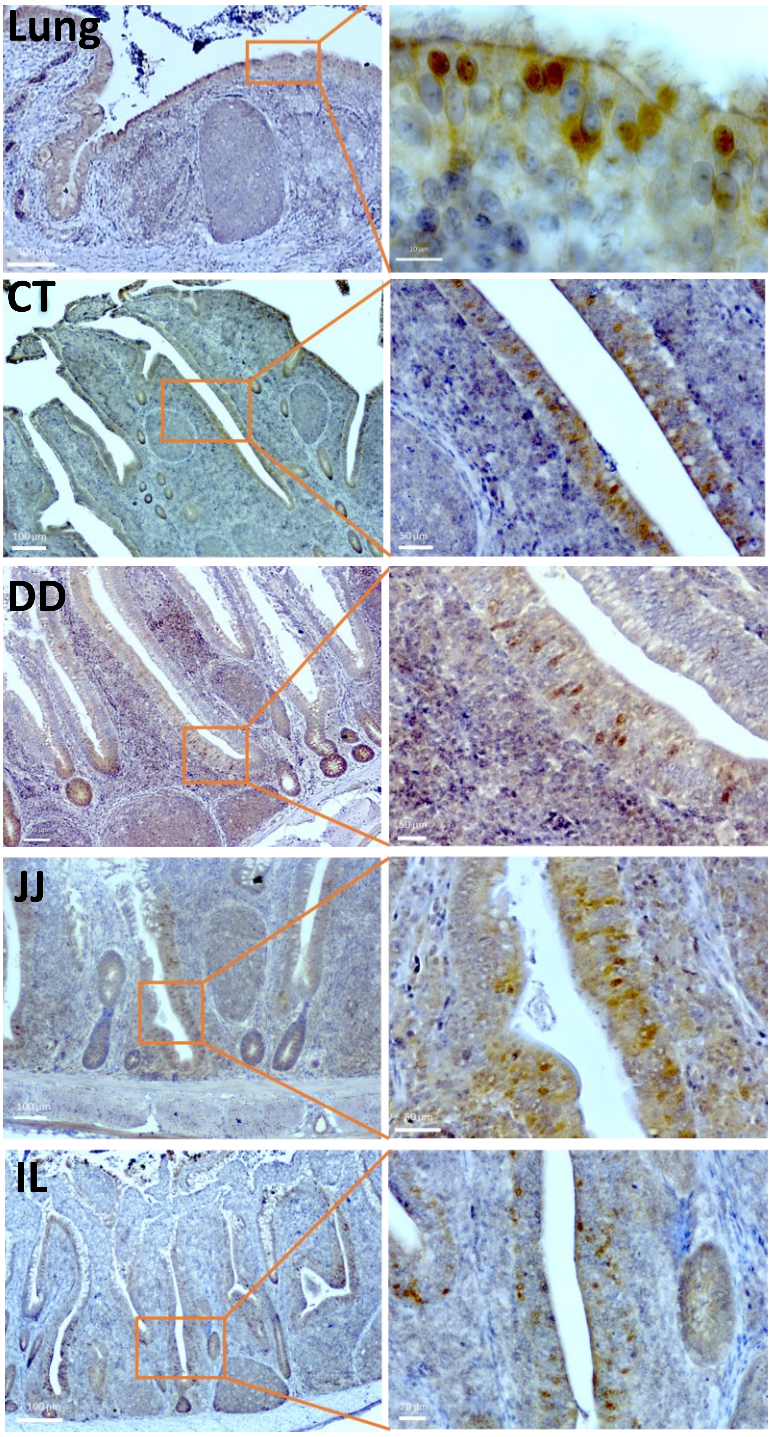

Supplementary Fig. 1. Expression of SOX8 in M cells of chicken BALT and small intestine. FFPE sections from lung, caecal tonsil (CT), and PP of the duodenum (DD), jejunum (JJ), and ileum (IL) were stained with rabbit anti-human SOX8 (brown nuclei) and nuclei were counterstained with haematoxylin. Data are representative of 3 biological replicates for duodenal, jejunal, and ileal PP and CT birds and 2 biological replicates for the BALT from 7-week-old *CSF1R*-reporter transgenic chickens.
